# Supplementary material for: CRISPR/Cas9-Mediated Mutagenesis of Sex-Specific Doublesex Splicing Variants Leads to Sterility in Spodoptera frugiperda, a Global Invasive Pest
Source: Cells. 2022 Nov 10;11(22):3557. doi: 10.3390/cells11223557 (PMC9688123; doi:10.3390/cells11223557)
Supplement: Supplementary file 1 [file cells-11-03557-s001.zip › cells-1999535-supplementary.pdf]

Supplementary Materials

Figure

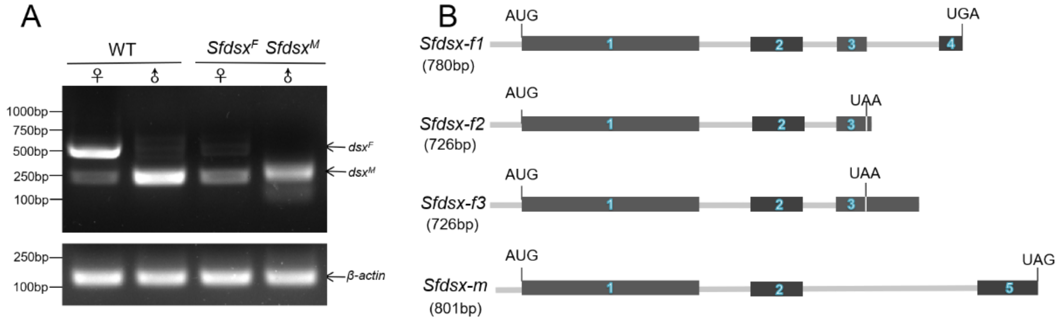

Figure S1. **Alternative splicing patterns of *Sfdsx* gene.** (A） RT-PCR was conducted to verify alternative splicing of *Sfdsx* gene in wild-type and *Sfdsx* mutant insects. The arrows indicate female- and male-specific splicing of *Sfdsx* flanking 500 and 250bp sequences respectively. The *ß-actin* gene was used as an internal control. (B) Genomic structure of *Sfdsx* gene. Four different alternative splicing patterns including three for females and one for male of sex-specific *Sfdsx* transcripts are shown. Exons are represented by boxes with labeling and intros are shown by lines.

Table

Table S1. Primers used in this study.

Name	Sequence (5'-3')	Purpose
dsx^{C}-sgF	TAATACGACTCACTATAGGGGTCCATATGTTCCCTG CGTTTTAGAGCTAGAAATAGCAA	sgRNA synthesis
dsx^{F}-sgF	TAATACGACTCACTATAGGGGAAATTAATAATATAA GGTTTTAGAGCTAGAAATAGCAA	
dsx^{M}-sgF	TAATACGACTCACTATAGGATTACGCAGGCAGTGA CGGTTTTAGAGCTAGAAATAGCAA	
sgR	AAAAGCACCGACTCGGTGCCACTTTTTCAAGTTGA TAACGGACTAGCCTTATTTTAACTTGCTATTTCTAGC TCTAAAAC	
dsx^{C}-site-F	AGCAGAGAACACTGATCCCTTA	Mutagenesis detection on genomic DNA
dsx^{C}-site-R	TTTGTACGAACGCTAAAAAGC	
dsx^{F}-site-F	CACGTTCCACACACAAAGTG	
dsx^{F}-site-R	AGACGGCAAACAAACGTCTC	
dsx^{M}-site-F	GTTTCACGCCAGCTTTCTT	
dsx^{M}-site-R	CTGCTTTGGCTCCTATTGAT	
β-actin-qF	CGGTATCGTGCTGGACTCCGGTG	Relative transcript analysis by RT-qPCR
β-actin-qR	GAGTAACCCCTCTCGGTGAGGATC	
dsx-qF	AAGCTGTTGGAGAAGTTCCACT	
dsx-qR	TATTTTCCGTGATGCCTCGT	
OR1-F	GCAGGCATGTTCAGAGATGA	
OR1-R	ACCCCATAGATGGAACACCA	
PBP1-F	ACGCTAGATGGAGGGTTGTG	
PBP1-R	CCGGTTGATGAGCTGGTACT	
PBP2-F	GCACAAGAATTTGCCATGAA	
PBP2-R	CACTTCTCCCACGACGAGTT	
dsx-cloneF	CTTAGTGGATAACTGTAACAAGCTG	Identification of sex-specific transcript
dsx-cloneR	GTACTCCGTGAAGCACATGG	
